# Clinical Outcomes and Return to Sports After Arthroscopic Repair of Humeral Avulsion of the Glenohumeral Ligament: A Meta-Analysis

**DOI:** 10.7759/cureus.40848

**Published:** 2023-06-23

**Authors:** Wael M Alzahrani, Nada F Tashkandi, Mawddah H Faqeeh, Wijdan S Almatrafi, Abdulaziz A Amer Bugnah, Abdullah H Kazim

**Affiliations:** 1 Department of Surgery, College of Medicine, Najran University, Najran, SAU; 2 College of Medicine, Almaarefa University, Riyadh, SAU; 3 Department of Family Medicine, College of Medicine, Umm Al-Qura University, Makkah, SAU; 4 College of Medicine, Umm Al-Qura University, Makkah, SAU; 5 College of Medicine, Najran University, Najran, SAU

**Keywords:** shoulder instability, hagl, meta-analysis, return to sport, arthroscopic repair

## Abstract

This study aimed to evaluate the clinical outcomes and the frequency of return to sport after the arthroscopic repair of a humeral avulsion of the inferior glenohumeral ligament (HAGL) lesion. Web of Science, Scopus, and Medline via PubMed and OVID were searched to identify the relevant citations. Screening and data extraction were performed independently. The Comprehensive Meta-Analysis software was used for all statistical analyses (CMA; USA version 3.3.070). A total of 18 articles (n = 832 patients; of whom, 379 patients had HAGL) were included. The fixed-effect estimate showed that the percentage of patients who returned to their sports was 89.1% (95% CI = 85% to 92.2%). The mean duration to return was estimated to be 6.65 months (95% CI = 5.10 to 8.20). Postoperatively, the mean Western Ontario Shoulder Instability Index (WOSI), Oxford Shoulder Instability Score (OSIS), and Subjective Shoulder Value (SSV) scores were 88.60 (95% CI = 86.18 to 90.98), 15.02 (95% CI = 7.42 to 22.63), and 86.90 (95% CI = 80.79 to 93.00), respectively. The Rowe score improved significantly postoperatively with a mean difference (MD) of 54.47 (95% CI = 39.28 to 69.66). The University of California - Los Angeles (UCLA) shoulder score increased significantly post-arthroscopic repair (MD = 10.91, 95% CI = 10.07 to 11.76). The current evidence suggests that arthroscopic repair of HAGL lesions is associated with a high percentage of return to sports and improved Rowe score, WOSI, UCLA shoulder score, OSIS scale, and SSV score. The quality of the included studies is moderate; however, these findings are promising and call for further multicenter, prospective studies.

## Introduction and background

Shoulder instability is often caused by humeral avulsion of the inferior glenohumeral ligament (HAGL), which is a rare cause with an estimated prevalence of 1-9% of traumatic shoulder instability cases [1-3]. Although HAGL can occur in isolation, it most often occurs in the presence of other pathologic entities, such as tears of the long head of the biceps tendon, rotator cuff tears, Hill-Sachs lesions, and Bankart lesions [3,4]. Abduction greater than 105 degrees coupled with external rotation has been linked to a failure of the capsule at the humeral attachment [5]. Other possible causes include high-energy trauma and recurrent microtrauma in overhead or throwing sports [6]. The inferior glenohumeral ligament (IGL) complex consists of an interposed axillary pouch and anterior and posterior bands; a lesion in these bands may result in anterior HAGL (AHAGL) or posterior HAGL (PHAGL). The size of the tear determines whether HAGL lesions need to be repaired or not. More anterior, anterior-inferior, inferior, and posterior glenohumeral translation has been shown in cadaveric biomechanical investigations of large HAGL lesions [7,8]. In contrast, shoulder translations and kinematics were not significantly affected by small HAGL lesions [8]. As a result, recurrent and ongoing shoulder instability may occur without treatment of large HAGL lesions [3,9]. Despite the importance of HAGL lesion repair, there is a lack of information on patient outcomes. To our knowledge, a few studies have compared the outcomes of open versus arthroscopic HAGL repair; nevertheless, case reports and small case series with a follow-up of 12-39 months indicate that both methods provide reliable shoulder stability in the short- and mid-term [10-12]. Wolf et al. showed that arthroscopic repair was associated with promising outcomes in patients with HAGL [2]. It is unknown how often concomitant lesions affect clinical outcomes after correcting the HAGL lesion. There is also a lack of reliable data that would enable us to predict whether or not a patient might safely return to sports after HAGL lesion repair. Therefore, this systematic review and meta-analysis aimed to summarize the current evidence regarding the clinical outcomes and the frequency of return to sport/work after the arthroscopic repair of a HAGL lesion.

## Review

Methods

This study has been reported in strict accordance with the Preferred Reporting Items for Systematic Reviews and Meta-Analyses (PRISMA) checklist and the Cochrane Handbook for Systematic Reviews of Interventions [13,14].

Eligibility Criteria

We included observational studies (case-control, cohort, case series, and cross-sectional) that reported data regarding the role of arthroscopic repair in patients with HAGL lesions. We excluded non-English-language studies and conference abstracts.

Information Sources and Search Strategy

We initiated a comprehensive database search on June 15, 2022, focusing on Web of Science, SCOPUS, and Medline through PubMed and OVID. Our search was centered around the following key terms: “Arthroscopic Repair,” “Arthroscopic Surgery,” “humeral avulsion of the Glenohumeral Ligament,” and “HAGL.” The search was conducted from the inception of these databases until the date of our search. Additionally, we scrutinized the reference lists of all gathered citations for any further relevant sources. To organize our findings and remove any duplicates, we employed EndNote X9 software.

Selection Process

We utilized Microsoft Excel to establish a screening spreadsheet that included details such as study ID, publication year, title, abstract, keywords, DOI, and URL. A two-tiered screening process was performed by three independent evaluators (NFN, MHF, and WSA). The first phase involved reviewing the titles and abstracts of all identified studies through the literature search to decide which ones might advance to the second phase. In the second phase, the full text of these shortlisted studies was scrutinized to ascertain if they met the eligibility requirements. Any discrepancies among the reviewers were resolved through the intervention of the study supervisor (WMA).

Data Items and Collection Process

An offline, pre-structured Excel sheet was used by four independent evaluators to capture the following information from the selected studies: patient demographics (specifically age and gender), features of the study (such as study groups, total sample size, country, and key findings), and outcomes. These outcomes included aspects such as return to sport, visual analog scale (VAS), Rowe score, University of California - Los Angeles (UCLA) Shoulder Scale, Simple Shoulder Test (SST) score, the score for the disability/symptom scale (Q-DASH), Western Ontario Shoulder Instability Index (WOSI) score, Oxford Shoulder Instability Score (OSIS), American Shoulder and Elbow Surgeons (ASES) score, Subjective Shoulder Value (SSV) score, and shoulder function measures, including external rotation, abduction, and flexion.

Risk of Bias and Quality Assessment

Two authors (AAB and AHK) independently conducted an assessment of the risk of bias and the quality of each selected article using the National Institutes of Health (NIH) quality assessment tool, which is suitable for observational cohort, case-control, and cross-sectional studies [15]. This tool aids reviewers in critically appraising the internal validity of the research. Based on this evaluation, studies were categorized as good, fair, or poor. If any discordance in ratings arose among the authors, a third author (WMA) stepped in to resolve the issue.

Data Synthesis

The percentage of return to sport was calculated using the fixed-model effect size at a 95% CI. The postoperative clinical outcomes were assessed using the pooled mean and 95% CI. When comparing preoperative and postoperative scores, the mean difference (MD) and 95% CI were computed. The degree of heterogeneity and discrepancy among studies was gauged using the I^2^ statistic, with values of 25%, 50%, and 75% regarded as low, moderate, and high heterogeneity, respectively. If the heterogeneity was substantial with I^2^ >50%, a random-effects model was applied; otherwise, a fixed-effect model was adopted. The Comprehensive Meta-Analysis software (CMA; USA: version 3.3.070) was utilized for all statistical computations. Publication bias was evaluated according to Egger’s test criteria, and a funnel plot was constructed for forest plots incorporating 10 or more studies. Statistical significance was ascribed to a p-value less than 0.05.

Results

Study Selection

**Figure 1:**
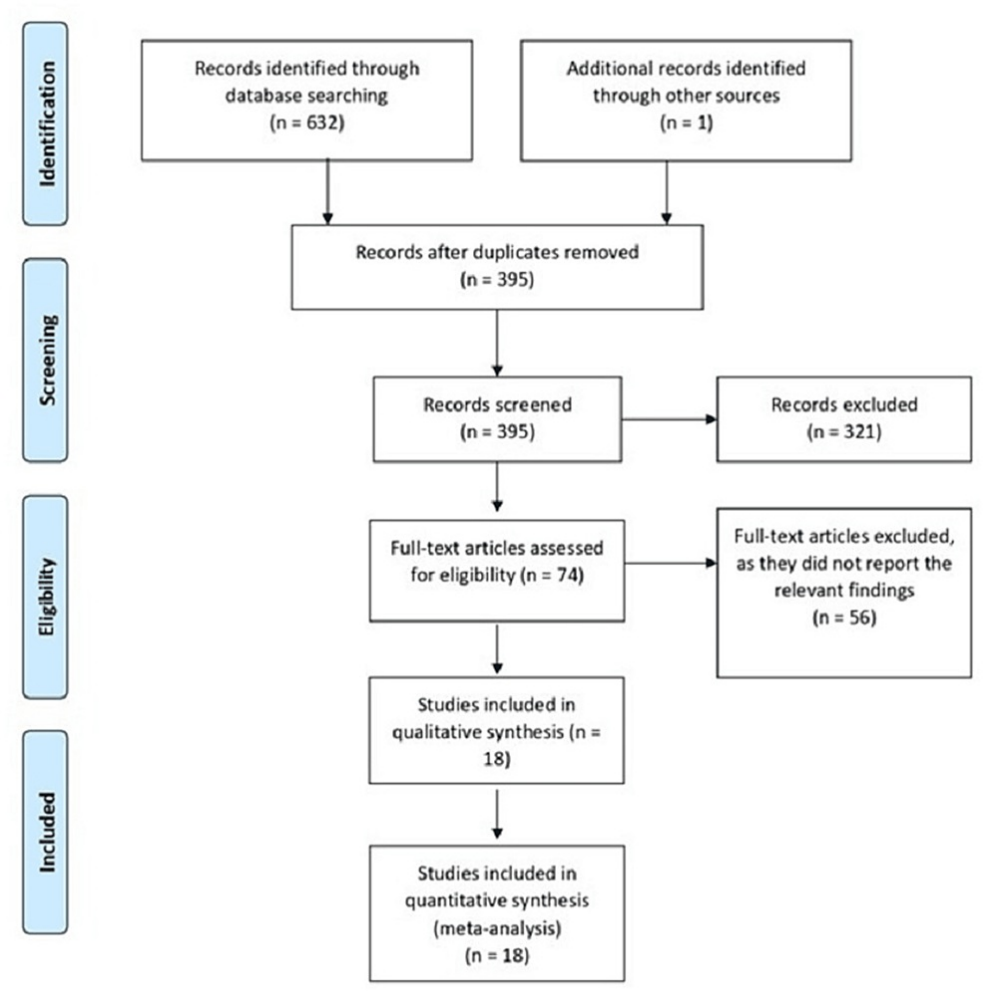
Preferred Reporting Items for Systematic Reviews and Meta-Analyses flow diagram.

Our literature search resulted in a total of 633 potentially relevant articles. After eliminating duplicates, we screened the titles and abstracts of 395 articles. Out of these, 321 were considered not suitable according to our criteria. We conducted a full-text review of 74 articles, of which 56 were subsequently excluded. Ultimately, we included 18 articles (representing a total of 832 patients; of them, 379 patients were diagnosed with HAGL) [2,16-32]. The PRISMA flowchart illustrating the process of inclusion of studies is presented in Figure 1.

Characteristics of Included Studies and Patients

Six studies were retrospective, five were prospective, six were case series, and one was a randomized clinical trial. Seven studies were conducted in the United States, two in the United Kingdom, two in Japan, two in Italy, one in Ireland, one in Brazil, one in Australia, and one in Switzerland, three in Italy, two in Australia, two in Germany, one in Brazil, and one in Japan. The included patients’ ages ranged between 15 and 35 years, and 55.77% were males. The follow-up period ranged from 1 to 3.5 years. The arthroscopic repair was conducted in 86% of the patients (Table 1).

**Table 1:** Summary of included studies. *: Of those who had humeral avulsion of the glenohumeral ligament. NA: not available; AR: arthroscopic repair; HAGL: humeral avulsion of the glenohumeral ligament

Study	Design	Country	Sample size	Age	Males	HAGL	AR	Rotator cuff lesions	Follow-up
Gulotta et al., 2014 [15]	Retrospective study	United States	5	33.5 years (31–37)	5 (100%)	5 (100%)	3 (60%)	3 (60%)	45.2 months (25–72)
Castricini et al. 2019 [29]	Prospective study	Italy	44	29.8 ± 8.9	42 (95%)	44 (100%)	6 (14%)	NA	29.6 ± 6.9
Schmiddem et al., 2019 [28]	Retrospective study	Australia	16	24 years	4 (25%)	16 (100%)	16 (100%)	5 (31.5%)	59 months (16–104)
Flury et al., 2016 [27)	Case series	Switzerland	8	31 years (19–63)	3 (37.5%)	8 (100%)	8 (100%)	1 (12.5%)	29 months (12–38)
Terra et al., 2013 [26]	Retrospective study	Brazil	12	28.9 years (18–45)	11 (91.67%)	12 (100%)	12 (100%)	1 (8.33%)	3.8 years
Davey et al., 2022 [25]	Retrospective study	Ireland	HAGL (n = 15)	21.5 ± 4.1	15 (100%)	15 (100%)	15 (100%)	3 (20%)	53.5 ± 17.4 months
			Control (n = 90)	21.2 ± 2.3	90 (100%)	-	90 (100%)	30 (33.3%)	55.1 ± 16.5 months
Nixon et al., 2015 [22]	Retrospective study	United Kingdom	57	16.8 ± 1	56 (98%)	1 (2%)	57 (100%)	NA	22 months
Taylor et al., 1997 [23]	Prospective study	United States	63	19.6 years	59 (93.65%)	63 (100%)	63 (100%)	0 (0%)	NA
Robinson et al., 2008 [24]	RCT	United Kingdom	88	24.3 ± 4.6	82 (91.18%)	88 (100%)	45 (51.14%)	0 (0%)	NA
DeBerardino et al., 2001 [21]	Prospective study	United States	48	20 (17–23)	45 (93.75%)	48 (100%)	48 (100%)	NA	37 (24–60)
Provencher et al., 2017 [20]	Prospective study	United States	27	24.9 (18–34)	15 (56%)	27 (100%)	10 (37%)	NA	36.2 (24–68)
Mizuno et al., 2005 [19]	Prospective study	Japan	303	25 (13–43)	9 (75%)*	12 (4%)	12 (100%)*	NA	NA
Grundshtein et al., 2021 [18]	Case series	Israel	23	24 years	11 (47.82%)	7 (30.4%)	11 (100%)*	NA	24.4 (7–99)
Chang et al., 2014 [31]	Case series	United States	4	28 ± 2.45	4 (100%)	4 (100%)	4 (100%)	2 (50%)	1 year
Taljanovic et al., 2011 [17]	Case series	United States	4	20.40 ± 0.69	0 (0%)	4 (100%)	4 (100%)	3 (75%)	NA
Kon et al., 2005 [30]	Case series	Japan	3	NA	2 (66.67%)	3 (100%)	3 (100%)	NA	NA
Wolf et al., 1995 [2]	Case series	United States	6	23.67 ± 10.15	4 (66.67%)	6 (100%)	4 (66.67%)	NA	39.83 ± 12.43
Castagna et al., 2007 [32]	Retrospective study	Italy	16	25.2 ± 3.2	7 (43.75%)	16 (100%)	16 (100%)	NA	34.2 months

Quality of the Included Studies

Based on the NIH quality assessment tool for observational studies, about 30% of the studies were deemed Good, 40% of the studies were considered Fair, and 30% of the studies were deemed Poor. The risk of bias in the randomized controlled trial was high.

Return to Sports

**Figure 2:**
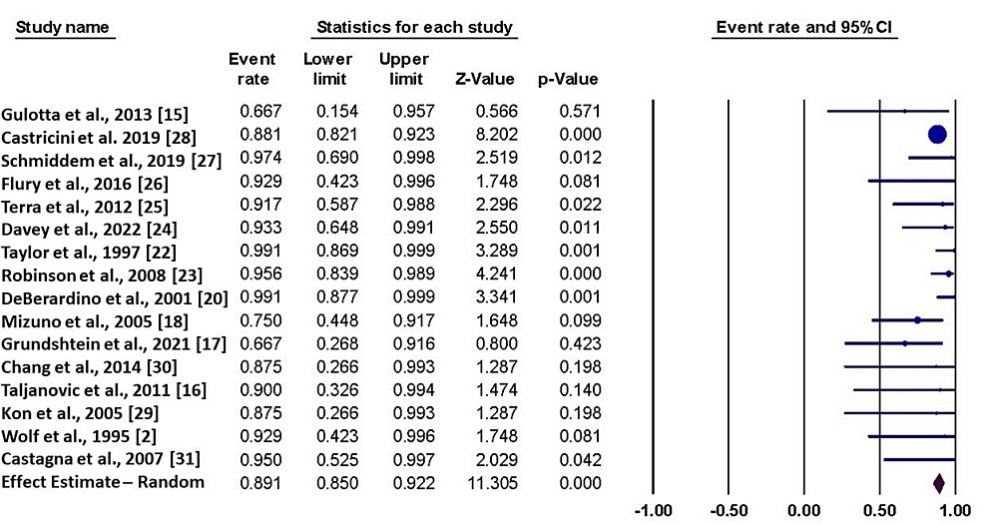
Forest plot of the return to sport. This figure shows the pooled analysis of 16 studies [2,15-18,20,22-31] regarding the return to sport.

Sixteen studies reported data about the return to sports. The fixed-effect estimate showed that the percentage of patients who returned to their sports was 89.1% (95% CI = 85% to 92.2%). The pooled data were homogenous (I^2^ = 11%, p = 0.32) (Figure 2).

**Figure 3:**
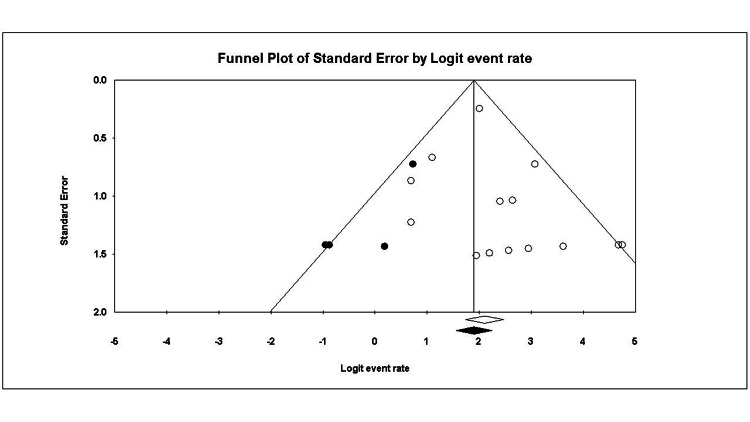
Funnel plot of the return to sport.

The funnel plot showed a potential risk of publication bias, which was not confirmed by the Egger test (p = 0.22) (Figure 3). The mean duration to return was estimated to be 6.65 months (95% CI = 5.10 to 8.20; I^2^ = 20%, p = 0.21).

Clinical Outcomes

Rowe score: Four studies reported the pre and postoperative Rowe score, with an MD of 54.47 (95% CI = 39.28 to 69.66). The pooled data were heterogenous (I^2^ = 80%, p = 0.002). The heterogeneity was resolved by excluding Grundshtein et al. (I^2^ = 0%, p = 0.70) with an effect estimate of (MD = 62.93, 95% CI = 59.28 to 66.59). Seven studies reported the postoperative Rowe score with a mean of 90 (95% CI = 85.9 to 94.02). The pooled data were moderately heterogeneous (I^2^ = 50%, p = 0.02). The heterogeneity was resolved by excluding Grundshtein et al. (I^2^ = 0%, p = 0.50) with an effect estimate of (mean = 89, 95% CI = 86.2 to 91.95).

VAS score: Two studies reported pre and postoperative VAS. The pooled effect estimate showed that arthroscopic repair was not associated with a reduced VAS score (MD = 0.01, 95% CI = -1.67 to 1.69). The pooled data were homogenous (I^2^ = 0%; p = 0.37).

UCLA shoulder score: The pooled analysis of two studies showed that the UCLA shoulder score increased significantly after arthroscopic repair (MD = 10.91, 95% CI = 10.07 to 11.76). The pooled data were homogenous (I^2^ = 0%; p = 0.61).

Shoulder function: The pooled analysis of two studies showed that the arthroscopic repair was not associated with improved external rotation, abduction, or flexion (MD = 10.15, 95% CI = -17.67 to 37.97; MD = 16.04, 95% CI = -11.18 to 43.27; and MD = 7.91, 95% CI = -19.31 to 35.13, respectively). The pooled analyses were homogenous in the three comparisons (I^2^ = 40%, p = 0.17; I^2^ = 0%, p = 0.73; and I^2^ = 0%, p = 0.77, respectively).

WOSI score: Two studies reported the postoperative score of WOSI. The pooled fixed-effect estimate demonstrated that the mean WOSI score postoperatively was 88.60 (95% CI = 86.18 to 90.98). The pooled data were homogenous (I^2^ = 0%; p = 0.424).

OSIS scale: Two studies reported the postoperative score of OSIS. The pooled fixed-effect estimate demonstrated that the mean OSIS score postoperatively was 15.02 (95% CI = 7.42 to 22.63). The pooled data were homogenous (I^2^ = 54%; p = 0.13).

SSV score: Two studies reported the postoperative SSV score. The pooled fixed-effect estimate demonstrated that the mean SSV score postoperatively was 86.90 (95% CI = 80.79 to 93.00). The pooled data were homogenous (I^2^ = 0%; p = 0.78).

Discussion

In this systematic review and meta-analysis, the current evidence suggests that arthroscopic repair of HAGL lesions was associated with a high percentage of return to sports and improved Rowe score, WOSI, UCLA shoulder score, OSIS scale, and SSV score. The quality of the included studies is moderate; however, these findings are promising and call for further multicenter, prospective studies.

Reconstruction of HAGL lesions has been documented using both arthroscopic and open methods, although only case reports and short series have been conducted. Although arthroscopic procedures are common, there may be substantial variation in how they are performed. A conventional posterior viewing portal is located roughly 2 cm inferior to the lower boundary of the posterolateral acromial angle and 2 cm lateral to the axillary pouch portal, which some surgeons prefer [33]. However, many advocate for a more anterior portal along the edge of the IGHL at the 5 o’clock position relative to the glenoid, around 1 cm inferior to the upper border of the subscapularis tendon, and as lateral as feasible [34]. In a study by Kon et al., three patients who underwent arthroscopic repair reported no symptoms and no recurrence of instability at a minimum 16-month follow-up [30]. Field et al. found similar findings after performing an arthroscopic repair on five patients with concomitant HAGL and Bankart lesions and following them for an average of 26 months [35]. Arthroscopic repair provides several benefits, including a shorter recovery time, a higher rate of success, a more aesthetically pleasing result, and improved function in the short term. However, there are several significant drawbacks, including technical complexity and the potential for axillary nerve damage [36].

The likelihood of a full recovery and restoration to pre-injury athletic performance following HAGL repair may be affected by the presence of other injuries and the intensity of the activity. Although the percentage of patients who returned to sports was very high, it was noted that not all patients returned to the same level. It was observed that patients who did not return to the same level of sports were associated with lower Rowe, OSIS, and ASES scores compared with those who returned completely [28]. Moreover, patients with concomitant rotator cuff injury or labral tear were less likely to return to the same level. It has been extensively reported that HAGL lesions often occur with other shoulder lesions [4]. Longo et al. conducted a systematic review of HAGL lesions and found that Hill-Sachs lesions accounted for 81% of all reported lesions, followed by anterior labral tears at 41% [6]. Other associated injuries, including bony Bankart lesions, posterior labral tears, and superior labral anterior-posterior tears, were described in the study; however, rotator cuff tears were not. Further research showed that 33%-36% of patients also had rotator cuff tears, and 15%-36% experienced labral tears [1,10,27]. Other variables, such as the fear of exerting severe effort and other socio-economic concerns, may impact the return to sports at the same level. Having limited external rotation may make it difficult to compete in shoulder-demanding sports. The findings, however, may largely be interpreted descriptively due to the small sample size.

Regarding the Rowe score, four studies reported a pre and postoperative Rowe score, with an MD of 54.47 (95% CI = 39.28 to 69.66) and a mean postoperative Rowe score with a mean of 90 (95% CI = 85.9 to 94.02). The Rowe score is considered excellent at 90 to 100 points, good between 89 and 75 points, fair between 74 and 51 points, and poor below 50 points [37]. These findings indicate that the arthroscopic repair of HAGL resulted in excellent stability, as assessed by the Rowe score. Similarly, in terms of ULCA shoulder score, our findings showed that the arthroscopic repair elevated it by about 11 points, with a postoperative mean score of 32.9, indicating better shoulder function. Nonetheless, compared to other outcome measures, the reliability, validity, and responsiveness of the UCLA shoulder score are not well-established [38]. The UCLA shoulder scoring system may be beneficial for some conditions, such as rotator cuff disease or shoulder instability. Researchers designing clinical studies, however, should use a more up-to-date tool created with adequate patient participation and proven validity and reliability [39].

The OSIS score is a patient self-completion patient-reported outcome measure with 12 items on daily living activities that are especially pertinent to patients with shoulder instability. The OSIS was created primarily to evaluate the effectiveness of treatment by monitoring patients’ pain levels and activities of daily life. In our study, the arthroscopic repair showed a significant elevation in the OSIS score, indicating that the level of daily living activities had improved; however, regarding the pain, the independent score of VAS demonstrated a non-significant improvement in managing the pain.

The WOSI score is a patient-reported outcome that assesses several quality of life domains, including physical symptoms, pain, sport, recreation, work, lifestyle and social functioning, and emotional well-being. The relatively high score of WOSI in our study highlights the significant improvement in the quality of life of these patients after arthroscopic repair. However, this score has some limitations, including the risk of recall bias, not sufficiently established validity and reliability, and the relatively extensive number of questions.

Even though this is the first meta-analysis to investigate the clinical outcomes of arthroscopic repair in patients with HAGL, we acknowledge that our study has some limitations, including the small sample size of included patients and studies, the moderate quality of evidence generated from the included studies, and the absence of head-to-head comparisons due to the lack of data.

## Conclusions

The current evidence suggests that the arthroscopic repair of HAGL lesions is associated with a high percentage of return to sports and improved Rowe score, WOSI, UCLA shoulder score, OSIS scale, and SSV score. The quality of the included studies is moderate. However, the study findings are promising and call for further multicenter, prospective studies.
